# Thermal and quantum depletion of superconductivity in narrow junctions created by controlled electromigration

**DOI:** 10.1038/ncomms10560

**Published:** 2016-02-16

**Authors:** Xavier D. A. Baumans, Dorin Cerbu, Obaïd-Allah Adami, Vyacheslav S. Zharinov, Niels Verellen, Gianpaolo Papari, Jeroen E. Scheerder, Gufei Zhang, Victor V. Moshchalkov, Alejandro V. Silhanek, Joris Van de Vondel

**Affiliations:** 1 Département de Physique, Université de Liège, B-4000 Sart Tilman, Belgium; 2 INPAC—Institute for Nanoscale Physics and Chemistry, Department of Physics and Astronomy, KU Leuven, Celestijnenlaan 200D, B-3001 Leuven, Belgium; 3 IMEC—Interuniversity Microelectronics Centre, Kapeldreef 75, B-3001 Leuven, Belgium; 4 Department of Physics, University Federico II of Naples, Piazzale Tecchio 80, 80125 Naples, Italy; 5 CNR-SPIN, Piazzale Tecchio 80, 80125 Naples, Italy

## Abstract

Superconducting nanowires currently attract great interest due to their application in single-photon detectors and quantum-computing circuits. In this context, it is of fundamental importance to understand the detrimental fluctuations of the superconducting order parameter as the wire width shrinks. In this paper, we use controlled electromigration to narrow down aluminium nanoconstrictions. We demonstrate that a transition from thermally assisted phase slips to quantum phase slips takes place when the cross section becomes less than ∼150 nm^2^. In the regime dominated by quantum phase slips the nanowire loses its capacity to carry current without dissipation, even at the lowest possible temperature. We also show that the constrictions exhibit a negative magnetoresistance at low-magnetic fields, which can be attributed to the suppression of superconductivity in the contact leads. These findings reveal perspectives of the proposed fabrication method for exploring various fascinating superconducting phenomena in atomic-size contacts.

## Abstract (web summary)

Nanostructured superconductors allow dissipationless electrical transport to be exploited in technologically relevant devices. Here, the authors follow how detrimental fluctuations of the superconducting order parameter evolve in Al atomic contacts as their width is controlled by electromigration.

## Graphical abstract

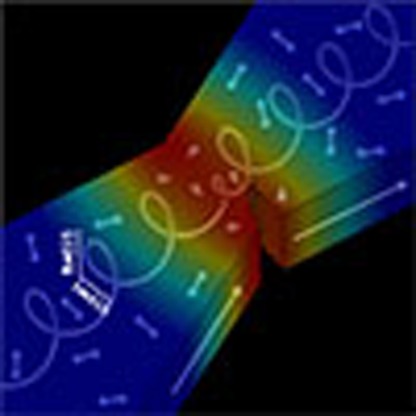

During the last decades, nanotechnology has set the stage for a new industrial revolution. Not only because it made possible the continuous miniaturization of larger devices, but mainly due to a plethora of unexpected emerging properties that have no bulk equivalent. When crossing over from macro, through the meso, for eventually reaching the microworld, we can identify two main physical properties that have an increasingly important role. First, surface effects arising from the increase of surface to volume ratio and the fact that surface atoms have a different coordination number than atoms in the bulk. Second, confinement effects, giving rise to a discretization of the electronic levels, a change of the density of states and the overall electronic properties^1^.

Superconducting materials at the nanoscale show no exceptions to this transformation. However, due to the multiple characteristic length scales of superconductivity, that is, magnetic penetration depth *λ*, coherence length *ξ* and Fermi wavelength *λ*_F_, nanoscale superconductors exhibit a far richer spectrum of phenomena^2^. More specifically, in superconducting devices with lateral dimensions ranging from 100 nm to a few micrometres (the so-called mesoscopic regime, size comparable to *ξ* and/or *λ*) strong confinement effects of the superconducting condensate are observed^3^. In even smaller nanostructures, with at least one dimension comparable to *λ*_F_, electron confinement effects come into play. As such, a profound size dependent effect is expected on the superconducting gap and the critical temperature, *T*_c_ (refs 4, 5).

In addition, while bulk superconductivity is characterized by a macroscopic wave function for the whole-Cooper pairs condensate 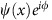 with a well-defined phase φ, in low-dimensional systems the long-range order of superconducting Cooper pairs is not possible due to phase fluctuations. As a consequence, a two-dimensional nanofilm goes through a phase transition from a superconducting state to an insulating state at low temperatures^6^. In a one-dimensional superconducting nanowire of cross section *S*<*ξ*(*T*)^2^, phase fluctuations (known as phase slips) lead to a premature suppression of the superconducting properties^7^,^8^,^9^,^10^. These phase slips represent activation processes that can be triggered either thermally^11^,^12^ or through quantum tunnelling^7^,^8^,^9^. As phase fluctuations cause dissipation, their growing importance with reducing dimensions seems to settle the lower limit for developing useful superconducting devices.

Although the aforementioned mesoscopic regime has been widely explored both experimentally and theoretically, the superconducting microworld has received much less attention. One of the main reasons for the limited amount of experimental results is the difficulty of sample fabrication, as conventional lithographic techniques are unable to reach these dimensions. As a result, researchers are exploring alternative approaches to develop controlled nano- and subnano-scale fabrication^7^,^13^,^14^,^15^. One promising direction here is a process known as electromigration (EM)^16^,^17^. This effect relies on the combination of local temperature rise and substantial current crowding at nanoconstrictions, which constitute the necessary ingredients to induce eventually a gradual displacement of atoms from their previously fixed position in the crystalline lattice. Uncontrolled EM is responsible for the breakdown of fine electronic interconnects. But when used in a controllable way, EM can serve as a mechanism to further decrease locally the cross section of the nanowire towards the single-atomic contacts regime^18^.

In this work, we investigate the conditions for nucleating phase slips in pre-indented Al nanowires, and the transition from thermally induced to quantum driven phase slips as the nanowires are narrowed down via *in-situ* controlled EM, that is, without the need to fabricate new samples. Our results are in agreement with the assumption that EM occurs at a constant power of 110 μW at the constriction, irrespective of the constriction size. We estimate that the transition from thermally activated phase slips (TAPS) to quantum phase slip (QPS) takes place at 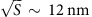, and we show that sufficiently narrow constrictions lead to a negative magnetoresistance (NMR), previously reported as a fingerprint of phase-slips-dominated dissipation^19^. We demonstrate that this negative magnetoresistance arises from the suppresion of the rate of activated phase-slips, as normal quasiparticle current is injected through the leads into the constriction. For a constriction with normal resistance about twice as much as the superconducting quantum resistance, we found clear signatures of a superconducting-to-insulator transition. These findings show the possibilities of EM to create and explore superconducting devices with ultimate small dimensions^4^.

## Results

### EM procedure

**Figure 1:**
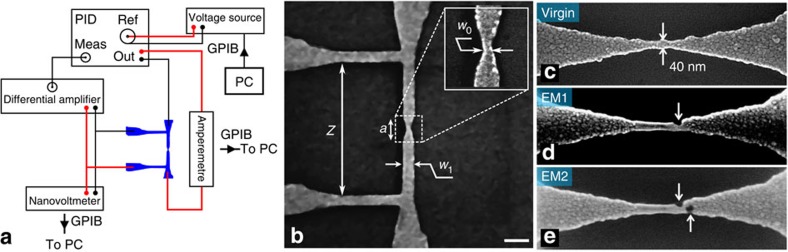
Electromigration set-up and layout of Al microbridges. (**a**) Schematic diagram of the combined analogue and digital feedback loop used to carry out controlled electromigration. Black and red lines correspond, respectively, to connections of negative and positive polarity. The connection between differential amplifier and PID controller is coaxial. Arrows from instruments indicate communication with the computer via general purpose interface bus (GPIB) interface. PID circuit has a reaction time of a few microseconds, whereas, GPIB-PC interface is in the millisecond range. (**b**) Representative scanning electron microscope (SEM) image of one of the samples investigated in this work, before electromigration. The bow-tie-shaped bridge is a thin film made of aluminium with dimensions: *Z*=2,000 nm, *a*=400 nm, *w*_1_=150 nm and *w*_0_=50 nm. Scale bar, 300 nm. (**c**–**e**) *Ex situ* scanning electron micrographs of a sample illustrating the shrinking of the constriction due to subsequent electromigrations. (**c**) corresponds to the virgin sample with arrows indicating the approximate width. (**d**) and (**e**) represents, respectively, the sample after the first and the second run of electromigration with arrows pointing out the created voids in the junction.

The schematic diagram of the experimental set-up is shown in Fig. 1a. To carry out highly controlled EM with little unwanted failures, we developed an electronic circuit which combines digital and analogue feedback similar to those reported in ref. 20. The standard procedure is as follows: the bias voltage is linearly ramped up in steps of few hundreds of μV every 100 ms while the resistance is constantly monitored. The role of the digital feedback loop is to maintain a change rate of the nanowire resistance (d*R*/d*t*) within certain predefined limits. The fact that the reaction of the digital circuit is relatively slow, thus likely causing breakdown of the structure, forces us to implement a secondary analogue feedback to damp ultra-fast overshoots of the resistance. Once the threshold resistance is reached, the voltage is automatically set to a very low value (a few μV). For more in-depth details of the circuitry and sample fabrication see the Methods section.

The Al nanowires fabricated by electron beam lithography have a constriction in the central part (bow-tie shape) as shown in the scanning electron microscopy image of Fig. 1b. *Ex situ* scanning electron microscopy images of a bow-tie sample before and after EM, Fig. 1c–e, clearly show that the constriction acts as nucleation point and guarantees that the EM will be triggered in its vicinity. We have investigated several different samples, with minor adjustments on the exact geometry, and all of them exhibit a quantitative correspondence between the normal-state resistance and its temperature dependence after repeated EM. Therefore, for the sake of clarity we will not explicitly indicate the sample source of each reported measurement.

The EM procedure was performed under two complementary cryogenic environments, namely a ^4^He cryostat with the sample immersed in liquid He and a ^3^He refrigerator with cooling achieved through a cold finger. EM carried out in the former system exhibits a higher degree of control and less unexpected breakdowns. This cryostat allows temperature control down to 100 μK accuracy and has a large cooling power. Its main disadvantage lies on the relatively high base-temperature of 1.1 K. EM performed in the ^3^He refrigerator seems to be less stable, the cooling power is limited to 50 μW and the temperature control to 5 mK accuracy, but the system offers a much wider temperature range (base temperature of 300 mK). Throughout this manuscript, measurements performed in both systems will be presented.

It is worth noting that the geometry of our sample is unambiguously known only before the first EM process takes place. For the bow-tie geometry used in this work, it is easy to derive the exact analytical formula for the resistance of the structure,


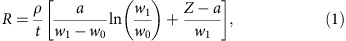


where *ρ* is the thin-film resistivity of Al, *t* is the thickness of the film, *w*_0_ is the width of the bridge at its narrowest point, *w*_1_ is the width of the bridge at its largest point, *a* is the length of the bridge where the cross section increases and *Z* represents the total length of the bridge (Fig. 1b). From this dependency, and knowing the exact geometry of our samples as well as their normal-state resistance *R*_n_=*R*(*T*>*T*_c_), we can estimate the resistivity of the Al bridges as *ρ*(1.5K)=5±1 μΩ cm. Using the relation *ρ*ℓ=4 × 10^−6^ μΩ cm^2^ valid for thin Al films^21^, we obtain an electronic mean free path ℓ=8 nm, that is, substantially smaller than any initial dimension of the bridge. We should point out that, for polycrystalline samples, differences up to a factor of 4 in *ρ*ℓ were reported^22^, meaning that the mean free path here estimated does not represent an accurate value but merely an order of magnitude. Nevertheless, the obtained mean free path is consistent with the rather small residual resistivity ratio *RRR*=*R*(300 K)/*R*(4.2 K)∼1.85±0.05, which indicates that our system falls in the diffusive transport regime.

**Figure 2:**
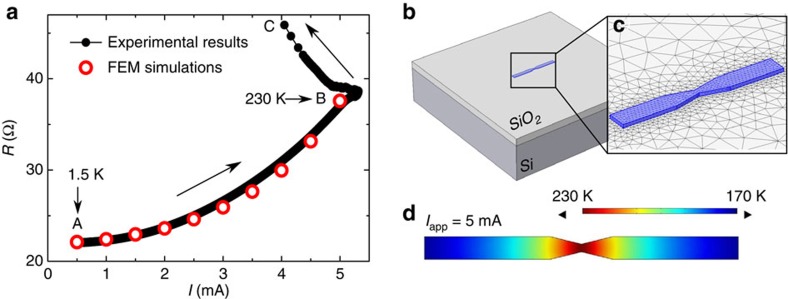
Inhomogeneous temperature distribution along the Al bridge before electromigration. (**a**) Non-monotonous resistance rise as a function of current during the first electromigration process. The solid black symbols represent the experimental data and open red dots are FEM simulations for a temperature coefficient of resistance given by *α*=3.6 × 10^−3^ K^−1^. Large black arrows indicate the temporal evolution of the acquired experimental data. Small arrows indicate the temperature of the junction at two points: at the beginning of the measurement (A) and at the onset of electromigration (B). (**b**) Al transport bridge on Si/SiO_2_ substrate immersed in liquid helium considered for FEM simulations. The sample dimensions are the same as for the real sample shown in Fig. 1 and the thickness of the SiO_2_ was taken as 100 nm. (**c**) Layout of the grid used for FEM simulations. (**d**) Simulated temperature profile of the bridge for an applied current of 5 mA.

In Fig. 2a, we show the resistance change observed during the first EM process (solid black symbols) in the ^4^He cryostat. The initial parabolic increase of resistance when increasing current results from a combination of two phenomena, (i) an inhomogeneous Joule heating and the consequent temperature rise, and (ii) a temperature dependent resistance. In other words, between points *A* and *B* in Fig. 2a, the *R*(*I*) curve does not follow an isothermal line, even if the sample is immersed in superfluid ^4^He. Furthermore, along this segment there is no EM, that is, no change of the initial geometrical shape in agreement with the reversible response observed when decreasing the current from any point in this region.

We can gain further insight about the initial virgin branch of the *R*(*I*) curve by performing finite element method (FEM) simulations using the commercial software COMSOL^23^. The layout of the modelled system, depicted in Fig. 2b, consists of a transport bridge lying on top of a SiO_2_/Si substrate all immersed in liquid helium at *T*=1.5 K. It has the exact same geometry as the experimentally measured one but without voltage contacts. The discrete grid of nodes with variable spatial density used for the FEM simulations is illustrated in Fig. 2c. The simulated Al sample has a temperature dependent resistivity given by the first-order Taylor expansion *ρ*(*T*)=*ρ*(1.5 K)[1+*α*(*T*−1.5 K)] with *ρ*(1.5 K) and *α*=d*ρ*/d*T*(=3.6 × 10^−3^ K^−1^) obtained experimentally. The results of the simulations shown by open symbols in Fig. 2a nicely reproduce the *R*(*I*) dependence followed by the experimental data. The small discrepancy between the experimental data and the numerical estimates most likely results from the assumption of a constant *α*. Moreover, the simulations allow us to obtain an estimate of the temperature along the parabolic segment *A*−*B*. In Fig. 2d, we show the temperature distribution along the transport bridge for *I*=5 mA, that is, just before starting the EM, indicated by point *B*. At this current value the temperature is as high as 230 K at the constriction. It is interesting to compare this value with previous reports. Indeed, Esen *et al*.^24^ estimated experimentally the temperature of the constriction during EM as ranging between 145 and 290 K. Independently, Trouwborst *et al*.^25^ obtained a *T*=400 K for the breaking process claiming that this value is rather independent of the temperature of the sample environment.

From point *B* till point *C* in Fig. 2a, a continuous EM occurs as evidenced by a decrease of the current in the feedback circuit with a consequent increase of sample resistance. From the moment that the first EM occurs the curve becomes irreversible and it is no longer possible to be certain about the geometry of the constriction. We have found that all the *B*−*C* segments corresponding to experimental data where EM is occurring, follow the very same law *R*≈*P*_c_/*I*^2^ from which a constant power of 110 μW was deduced. This value is similar to that previously reported in the literature for Au structures^18^.

### TAPS to QPS transition for electromigrated constrictions

Let us now investigate the evolution of the superconducting state in the electromigrated nanowires. More precisely, in this section we will study the development of phase fluctuations in constrictions with cross sections controlled by EM.

**Figure 3:**
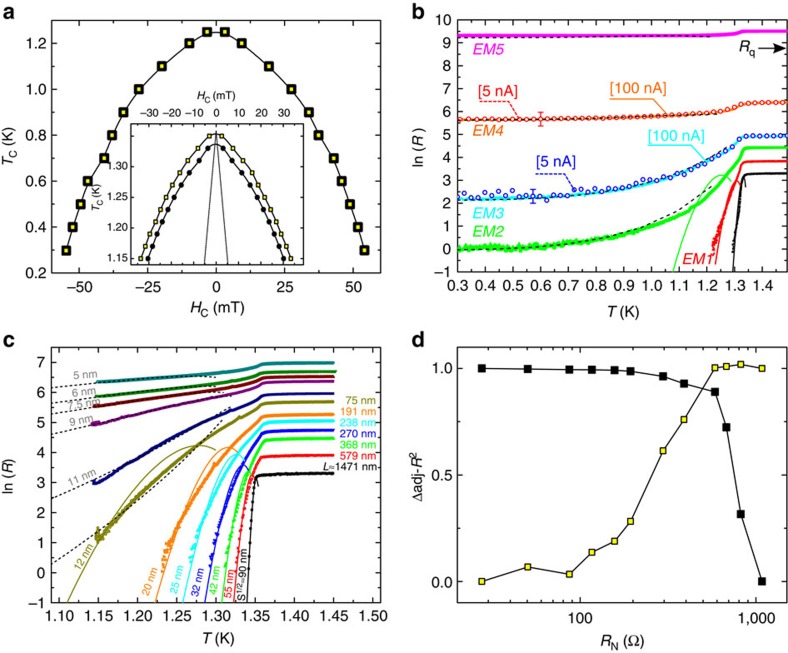
Transition from thermal to quantum phase slips. (**a**) Phase boundaries of the virgin sample. Inset: comparison of the phase boundary before (yellow squares) and after 16 runs of electromigration (black circles). The dotted line indicates the expected phase boundaries for bulk aluminium. (**b**) Logarithm of the resistance as a function of temperature after several electromigrations. The same curves are obtained for *I*=100 nA (continuous lines) as for *I*=5 nA (circles). For the sake of clarity, the curves taken at 5 nA show a limited amount of points. The error bars represent the standard deviation around the mean value obtained by averaging 200 data points. Black arrow indicates the position of the superconducting quantum resistance, *R*_q_. (**c**) All the successive *R*(*T*) curves after each run of electromigration. The cross section of the constriction (*S*) and the length (*L*) after each electromigration are obtained as adjusting parameters of the model. In panels (**b**) and (**c**), the continuous lines are fits using equation 3 for TAPS. The dashed lines are fits by the Golubev–Zaikin model for QPS. (**d**) Normalized adjusted-R^2^ (goodness of fit parameter), as a function of the resistance in the normal state. Yellow squares represent the fits using TAPS while black squares denote the fits using QPS.

Figure 3a shows the superconductor-normal metal phase boundary determined by a criterion of 0.9R_N_ in a virgin sample down to 300 mK and for magnetic fields applied perpendicularly to the plane of the sample. The inset in this panel shows the high-temperature part of *H*−*T* phase boundary for a virgin sample and for the same sample after 16 EMs. It is interesting to note that besides a vertical shift towards lower *T*_c0_ (critical temperature at zero field) caused by the broadening of the *R*(*T*) in the EM sample, both phase transition lines are very similar. These lines do not follow the linear phase boundary expected for an Al plain film (black line in the inset of Fig. 3a) but rather exhibit a parabolic dependence characteristic of small-dimension bridges (*w*<*ξ*(*T*)) given by^3^,^26^,^27^,


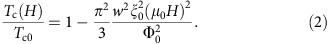


The fact that the phase boundaries before and after EM do not show significant differences suggests that the constriction has little influence on the nucleation of superconductivity, which probably takes place first along the leads. Based on this consideration, it is reasonable to take *w*=*w*_1_ in equation 2, which gives a value for the superconducting coherence length of *ξ*(0)=85±5 nm. This value is substantially smaller than the Pippard coherence length (*ξ*_0_≈1,600 nm) thus indicating that our nanostructured superconductor falls in the dirty limit. In this case, *ξ*(0)=0.855(*ξ*_0_ℓ)^1/2^ and we can estimate ℓ≈6 nm which is consistent with the value obtained from the normal-state resistivity.

As anticipated above, the shifting of the phase boundary after EM arises from a broadening of the superconductor-normal transition. This is more apparent in Fig. 3b which shows *R*(*T*) curves after subsequent EMs. It is evident from this figure that after EM, the normal-state resistance increases as a consequence of the cross section reduction at the constriction, and the superconducting transition broadens.

There are several sources of *R*(*T*) broadening that should be considered separately and in detail. First of all, the widening could result from the fact that a too high current has been used during the measurements. Therefore, as the constriction shrinks, for the same applied current, the current density increases after each EM and so does the dissipation^28^. To rule out this possibility, we have also acquired *R*(*T*) curves for much smaller current values. In Fig. 3b, we plot an example of two *R*(*T*) curves measured after EMs, for alternating current *I*=5 nA (open symbols) and *I*=100 nA (solid symbols). It can be clearly seen that decreasing the current has no major effect besides increasing the noise of the measured curve which is indicated by an error bar in Fig. 3b. Another possible cause of transition broadening could be the local heating at the constriction. This effect can also be disregarded based on the current independent result shown in Fig. 3b.

It has been recently recognized by Zgirski *et al*.^29^ that inhomogeneities along the nanowire might lead to broad superconducting transitions. In our particular case, the bow-tie bridge should be regarded as inhomogeneous in width. However, the *R*(*T*) transition at zero magnetic field for the virgin sample is rather abrupt (∼50 mK). In addition, since thinning the superconductor leads to a reduction of the effective mean free path which in turn results in an increase of *T*_c_, we expect a broadening caused by an increase of *T*_c_ (ref. 29). This is clearly not the case, since we observe the same *T*_c_ irrespective of the size of the constriction. Moreover, inhomogeneities along the wire cannot explain a finite dissipation below 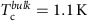, as already pointed out by Zgirski *et al*.^29^.

A more plausible explanation for the *R*(*T*) broadening is the excitation of phase slips at the constriction. Indeed, as originally proposed by Langer and Ambegaokar^11^, the phase of the superconducting order parameter can slip by 2*π* generating a normal region of core size ξ which gives rise to an excess of quasiparticles over a larger length scale 2Λ_Q_>>*ξ*, known as charge-imbalance length^11^. When triggering of phase slips is thermally activated^11^,^12^, it can be described by the formula following the theory of Lamber–Ambegaokar–McCumber–Halperin:


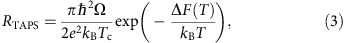


where Ω=[*L*/*ξ*(*T*)][Δ*F*(*T*)/*k*_B_*T*]^1/2^[1/*τ*_GL_] is the attempt frequency, 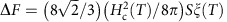 is the energy barrier for phase slips, *τ*_GL_=[*πℏ*/8*k*_B_(*T*_c_−*T*)] is the characteristic relaxation time in the time-dependent Ginzburg–Landau theory, 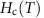 is the thermodynamic critical field, *k*_B_ the Boltzmann constant and *ξ*(*T*) is the coherence length. After each EM, the cross section of the constriction, *S*, and as a consequence the barrier Δ*F*, are reduced, thus increasing the nucleation frequency of phase slips.

In Fig. 3c we have made an attempt to fit all the *R*(*T*) transitions at high temperatures by only adjusting the parameters *S* and *L* which are explicitly indicated in the figure. The fitting is relatively good for the low-temperature part of the curves, but only for the first six EMs. The model clearly fails to follow the progressive change of concavity of the *R*(*T*)'s for the subsequent EM. This effect is captured by the parameter normalized adjusted-*R*^2^ representing the goodness of fit, as shown in Fig. 3d with square black symbols. A similar procedure was used to fit the *R*(*T*) curves shown in Fig. 3b covering a much wider temperature range. Here also the TAPS model only accounts for the fast drop of resistance with decreasing temperature for curves with low normal-state resistance, that is, wide constrictions.

A simpler alternative to the Lamber–Ambegaokar–McCumber–Halperin model (equation 3) with a temperature independent pre-factor has been proposed by Bezryadin *et al*. in ref. 30. Following this approach does not change significantly the value of the effective cross sections deduced from the fitting. It has been also proposed by Newbower *et al*.^31^ to take into account the parallel conduction channel provided by normal quasiparticles near *T*_c_. By including this model, we did not note significant improvements in the fittings or changes for the values of *S* and *L*.

A natural explanation for the observed breakdown of the TAPS model comes from the fact that once the barrier Δ*F* has been sufficiently reduced, tunnelling through, instead of surmounting it, becomes more likely. This regime of QPS has been theoretically investigated by Golubev–Zaikin who predicted a temperature dependence of the resistance given by^8^,


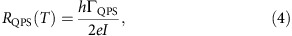


where


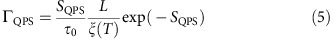


is the rate of QPS activation, *τ*_0_∼*h*/Δ is the characteristic response time of the superconductor, 
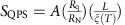
 is the effective action, *R*_q_=*h*/4*e*^2^, *R*_N_ is the resistance in the normal state, *ξ*(*T*) is the coherence length, *L* is the length of the wire and *A* is a numerical constant. In this formula, all values are known except A and L.

In Fig. 3b,c we have shown with dashed black lines the fitting corresponding to the QPS model using as free parameters: *A* and *L*. We can clearly see that the QPS fit works well where the TAPS model fails. This is also reflected in the goodness of fit parameter shown in Fig. 3d with yellow squares. From these fits, values of *L*≈85±10 nm and *A*≈1.1±0.1 are obtained. By using alternative formulas, one coming from a heuristic argument by Giordano^32^ echoed, among others, by Lau *et al*.^9^ and Altomare *et al*.^33^ and the other used by Bae *et al*.^30^, we obtained very similar results. The disadvantage of these approaches is that they contain two additional numerical fitting constants.

As a warning note we would like to emphasize that the presented analysis should be considered as purely qualitative though clearly suggesting that a transition from thermal to quantum excitation of phase slips at the nanoconstriction takes place. The *S* values obtained from the TAPS fitting should be taken with precaution and are only given (as well as *L*) for 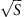 values higher than ∼12 nm, since the model clearly fails to adjust the tendency from and below that value. It is remarkable that this transition value is in good agreement with those previously reported in the literature. Zgirski *et al*.^28^ estimated a crossover at 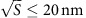 for the appearance of QPS whereas Bezryadin *et al*.^7^ reported 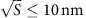. The values of 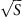 given in light grey for the high-resistance curves in Fig. 3c are indicative as they were extrapolated from the previous values of *S* as a function of *R*_N_. Indeed, *S*(*R*_N_) plot in logarithmic scales has been found to be clearly linear.

**Figure 4:**
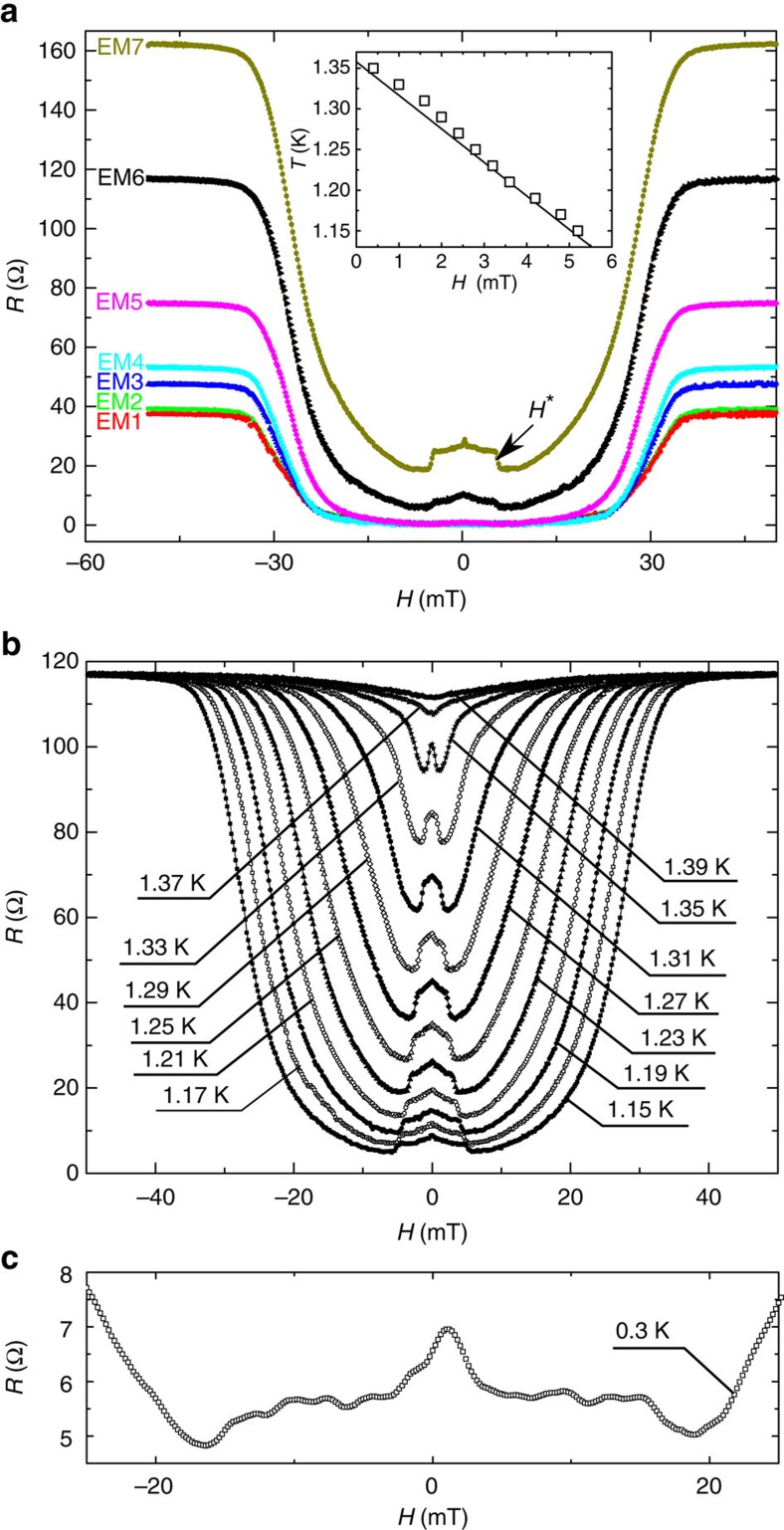
Negative magnetoresistance. (**a**) Resistance as a function of applied magnetic field at 1.15 K, for one-single sample after each run of EM. The inset shows the position of the NMR transition, H*, as a function of temperature (black empty squares). The black line corresponds to *H*_c2_ of the contact leads. (**b**) Resistance as a function of applied magnetic field at different temperatures between 1.15 and 1.39 K increasing by steps of 0.02 K. These multiple *R*(*B*) curves were taken just after the black curve (EM6) of **a**. (**c**) illustrates the persistance of the negative magnetoresistance effect down to 300 mK, where the electric response is dominated by QPS.

Another important evidence for the presence of phase slips comes from the development of a NMR effect as shown in Fig. 4a. Indeed, it has been shown experimentally and modelled theoretically that the presence of phase slips (TAPS or QPS) in superconducting nanowires leads to an unusual NMR for sufficiently narrow bridges. As of today, the origin of this effect is not yet fully understood. A possible mechanism could be the suppression of the rate of activated phase slips as a consequence of the decrease of the charge-imbalance length *Λ*_Q_ with magnetic field^34^,^35^,^36^,^37^,^38^.

An alternative proposed model suggests that the NMR arises from the suppression of superconductivity in the leads. This effect causes an increase of the normal component of the current (*I*_N_) running through the bridge and consequently a decrease of the rate of appearance of phase slips (triggered by the superconducting current *I*_S_)^34^. This second mechanism could lead to the observed sharp transition (corresponding to the destruction of superconductivity by the applied magnetic field in the leads). If this were the case, the limit of the NMR should coincide with the position of *H*_c2_ of the plain film. In Fig. 4b we show the evolution of the plateau as a function of temperature and in the inset of Fig. 4a, we show the position of the NMR transition (with a 50% criterion) compared with the phase boundaries obtained for the Al leads. The clear correspondence between destruction of superconductivity in the leads on the one hand, and NMR transition on the other hand, seems to reinforce this interpretation^19^. It should also be noted that this interpretation is consistent with our sample dimensions, considering that the value of the charge-imbalance length (*Λ*_Q_) in aluminium is situated around a few micrometres^39^,^40^,^41^. Fig. 4c shows the presence of the NMR at the lowest accessible temperature, in the regime dominated by the QPS.

**Figure 5:**
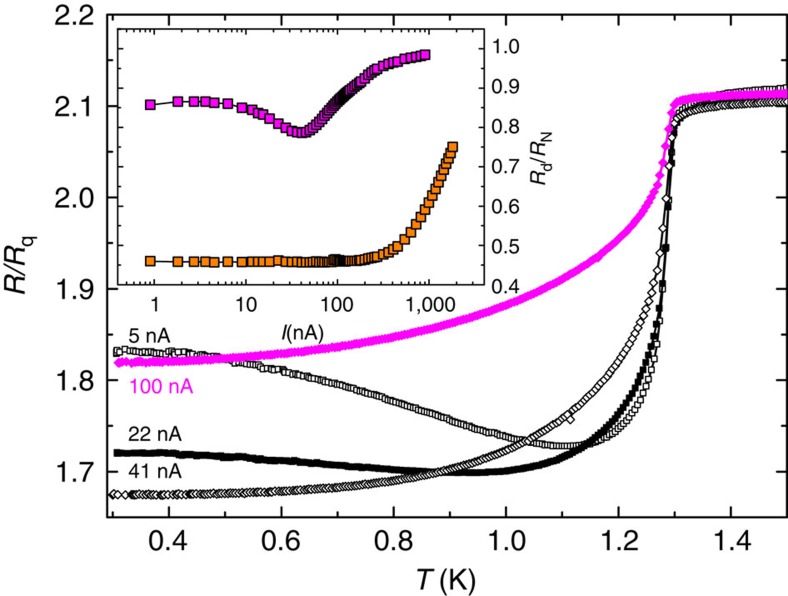
Superconducting to insulator transition. Resistance versus temperature at different currents for a constriction with *R*_N_>*R*_q_. At low currents and temperatures, *dR*/*dT*<0 is characteristic of a insulating state. The inset shows that for *R*_N_<<*R*_q_ (orange squares), the differential resistance is rather independent of the applied current, whereas for *R*_N_>*R*_q_ (purple squares), the differential resistance maximizes at zero bias. Measurements presented in the inset were taken at a temperature of 300 mK.

The measurements performed in a sample with *R*_N_>*R*_q_ unveiled further interesting results. Indeed, once the normal-state resistance of the electromigrated sample increases above the quantum resistance (indicated by a black arrow in the Fig. 3b), the adjustment with the QPS model no longer reproduced the *R*(*T*) shape at low temperatures. This behaviour, similar to a superconductor-insulator transition, has been already reported in ultra thin nanowires by Bezryadin *et al*.^7^ and attributed to a dissipative quantum phase transition^42^,^43^ analogous to the Schmid phase transition^44^ observed earlier in Josephson junctions^45^. Notice that, as mentioned in ref. 8, a dissipative quantum phase transition can be observed only provided that QPS are easily created at the constriction. Furthermore, unlike the superconducting state where d*R*/d*T* is always positive irrespective of the current value or temperature, the insulating phase is characterized by a negative d*R*/d*T* at low temperatures, as shown in Fig. 5 for the lowest values of the applied current. In addition, a differential resistance *R*_d_=d*V*/d*I* decreasing with increasing current, as shown in the inset of Fig. 5, represents also a signature of superconductor to insulator transition. Curves in this inset correspond to measurements done after EM4 and EM5 on the sample of Fig. 3b, as it is highlighted by the corresponding colours.

## Methods

### Samples fabrication

The Al nanowires were defined by electron beam lithography on Si substrate (275±25 μm) covered by a resist mask (single-layered, 3% PMMA 950 K in chlorobenzene solvent). The Si wafer had crystal orientation <100>, was p-doped (with Boron at concentrations 10^16^–5 × 10^14^ atoms per cm^3^) and has a native oxide layer (SiO_2_) on top (∼10 Å). Subsequently, an Al thin film (∼25 nm) was deposited using molecular beam epitaxy with deposition rate of 1.2 Å s^−1^ and pressure in the chamber under 10^−8^ mbar. Deposition was subsequently followed by lift-off process.

### Controlled EM

EM was basically achieved by applying a soft ramp of voltage to our samples. As you can see in Fig. 1a, a proportional-integral-derivative (PID) controller was used for analogue feedback: the voltage across our sample was constantly and fast monitored by a differential amplifier which gives its value to the PID controller so it can regulate the current in our sample. A computer running home-made software was used in the digital feedback loop: values of the resistance of our sample were deduced from the measurements of a nanovoltmeter (more accurate but slower than the amplifier) and the amperemeter. Then the computer chooses to increase/decrease the voltage to keep d*R*/d*t* in a pre-set range of values.

### FEM simulations

To model temperature fields and corresponding resistance of the nanowires, we used commercial FEM software COMSOL^23^. A electrical current density was simulated through the wire, substrate was considered electrically insulating. The whole sample was immersed in liquid helium (temperature of 1.5 K). The electrical resistivity of aluminium was chosen to be 3.6 μΩ cm. The thermal conductivity for aluminium, Si and SiO_2_ were assigned to 160 W m^−1^ K^−1^, 130 W m^−1^ K^−1^ and 1.4 W m^−1^ K^−1^, respectively. Heat transfer coefficient for convective cooling had a value of 30,000 W m^−2^ K^−1^.

## Additional information

**How to cite this article:** Baumans, X. D. A. *et al*. Thermal and quantum depletion of superconductivity in narrow junctions created by controlled electromigration. *Nat. Commun.* 7:10560 doi: 10.1038/ncomms10560 (2016).
